# L1 cell adhesion molecule overexpression in hepatocellular carcinoma associates with advanced tumor progression and poor patient survival

**DOI:** 10.1186/1746-1596-7-96

**Published:** 2012-08-13

**Authors:** Xiaodong Guo, Lu Xiong, Lin Zou, Ting Sun, Jing Zhang, Hanwei Li, Ruiyun Peng, Jingmin Zhao

**Affiliations:** 1 Postgraduate Medical School of PLA, Beijing, 100853, China; 2 302 Hospital of PLA, Beijing, 100039, China; 3 Beijing Institute of Radiation Medicine, 27 Taiping Road, Beijing, 100850, China; 4 PLA GENERAL HOSPITAL Beijing China, Beijing, 100853, China; 5 Navy General Hospital of PLA, Beijing, 100049, China

**Keywords:** Hepatocellular carcinoma, L1 cell adhesion molecule, Expression, Tumor progression, Prognosis

## Abstract

**Objective:**

L1 cell adhesion molecule (L1CAM), as a member of the immunoglobulin superfamily, has recently been observed in a variety of human malignancies. However, no data of L1CAM are available for hepatocellular carcinoma (HCC). The aim of this study was to investigate the expression of L1CAM in HCC and determine its correlation with tumor progression and prognosis.

**Methods:**

One-hundred and thirty HCC patients who had undergone curative liver resection were selected and immunohistochemistry, Western blotting, and quantitative real time polymerase chain reaction (Q-PCR) were performed to analyze L1CAM expression in the respective tumors.

**Results:**

Immunohistochemistry, Western blotting, and Q-PCR consistently confirmed the overexpression of L1CAM in HCC tissues compared with their adjacent nonneoplastic tissues at both protein and gene level (both P <0.01). Additionally, the high expression of L1CAM was significantly associated with advanced tumor stage (P = 0.02) and advanced tumor grade (P = 0.03), respectively. Moreover, HCC patients with high L1CAM expression were significantly associated with lower 5-year overall survival (P <0.01) and lower 5-year disease-free survival (P <0.01), respectively. The Cox proportional hazards model further showed that L1CAM over-expression was an independent poor prognostic factor for both 5-year disease-free survival (P = 0.02) and 5-year overall survival (P = 0.008) in HCC.

**Conclusion:**

Our data suggest for the first time that L1CAM expression in HCC was significantly correlated with the advanced tumor progression and was an independent poor prognostic factor for both overall survival and disease-free survival in patients with HCC.

**Virtual slides:**

The virtual slide(s) for this article can be found here: http://www.diagnosticpathology.diagnomx.eu/vs/1970024872761542

## Introduction

Hepatocellular carcinoma (HCC) is one of the most frequent human malignancy worldwide. Especially in China, it has become a major cause of cancer-related death [1]. There are many risk factors associated with HCC disease etiology, such as hepatitis B virus (HBV) and hepatitis C virus (HCV) infection, genetic makeup and environmental exposure [2]. The main features of HCC are fast infiltrating growth, early metastasis, high-grade malignancy, and poor therapeutic efficacy. Although surgical resection is the best method to ensure long-term survival for HCC patients, frequent recurrence and a high incidence of metastasis often make the overall prognosis unsatisfactory. The progression of HCC is associated with cumulative genomic alterations, including oncogene upregulation and tumor suppressor downregulation. Therefore, it is necessary to clarify the molecular pathogenesis of HCC.

The L1 cell adhesion molecule (L1CAM, also named as CD171), as a member of the immunoglobulin superfamily, is a 220 kDa type I transmembrane glycoprotein [3]. It is comprised of six IgG-like domains and five fibronectin-type III repeats, followed by a transmembrane region and a highly conserved cytoplasmic tail [4]. L1CAM was first described in neural cell migration [5]. Then, several studies demonstrated that this protein plays pivotal roles in mediating the correct formation of neuronal connections during embryo neurogenesis and performs important functions in neuron-neuron adhesion, neurite fasciculation, synaptogenesis, neurite outgrowth on Schwann cells, and neuronal cell migration [6-8]. In other normal cells such as hematopoietic, endothelial, and intestinal crypt cells, L1CAM is also detected, however, its function is unclear [9,10]. Recent studies have found the association of L1CAM expression with various human malignancies by analysis using established cell lines, and fresh frozen or fixed paraffin-embedded tissues. As the results, L1CAM expression was observed in lung cancer cell lines, gallbladder carcinomas, colon cancer, pancreatic cancer, renal cell cancer, gliomas, ovarian carcinomas, endometrial carcinomas, and melanomas [11-15]. However, no data of L1CAM are available for HCC. The aim of this study was to investigate the expression of L1CAM in HCC and determine its correlation with tumor progression and prognosis.

## Materials and methods

### Patients and tissue samples

The study was approved by the Research Ethics Committee of 302^nd^ Hospital of PLA, Beijing, China. Informed consent was obtained from all of the patients. All specimens were handled and made anonymous according to the ethical and legal standards.

A total of 130 patients with primary HCC who underwent a curative liver resection at the 302^nd^ Hospital of PLA, Beijing, China, were included in this retrospective study. Tissues used in the study were retrieved from the tissue bank of the Department of Pathology in the 302^nd^ Hospital of PLA. These patients were diagnosed as HCC between 2001 and 2006. None of the patients recruited in this study had chemotherapy or radiotherapy before the surgery. HCC diagnosis was based on World Health Organization (WHO) criteria. Tumor differentiation was defined according to the Edmondson grading system. Liver function was assessed using the Child-Pugh scoring system. Tumor staging was determined according to the sixth edition of the tumor-node-metastasis (TNM) classification of the International Union against Cancer. No distant metastasis was present at the time of surgery. The clinicopathological features of 130 patients are summarized in Table 1.

**Table 1:** Clinicopathological features and the expression of L1CAM in 130 HCC patients

**Clinicopathological Features**	**Case**	**L1CAM expression frequency (n,%)**		**P**
		**High**	**Low**	
**Age (years)**				
≤50	72	45 (63.13)	27 (23.61)	0.6
>50	58	37 (63.79)	21 (25.86)	
**Gender**				
Male	96	62 (64.58)	34 (35.42)	0.5
Female	34	20 (58.82)	14 (41.18)	
**Tumor stage**				
T1	23	5 (21.74)	18 (78.26)	
T2	40	12 (30.00)	28 (70.00)	0.02
T3	52	50 (96.15)	2 (3.85)	
T4	15	15 (100.00)	0 (0)	
**Tumor grade**				
G1	31	4 (12.90)	27 (77.10)	
G2	76	55 (72.37)	21 (27.63)	0.03
G3	23	23 (100.00)	0 (0)	
**Growth pattern**				
Trabecular	101	62 (61.39)	39 (38.61)	0.6
Nontrabecular	29	20 (68.97)	9 (31.03)	
**Cirrhosis**				
Yes	86	56 (65.12)	30 (34.88)	0.5
No	44	26 (59.09)	18 (40.91)	
**Underlying liver disease**				
Alcoholic	25	18 (72.00)	7 (28.00)	
Hepatitis B	49	40 (81.63)	9 (18.37)	0.3
Hepatitis C	35	12 (34.29)	23 (65.71)	
Unknown	21	12 (57.14)	9 (42.86)	

The median follow-up period was 8.6 years. Postoperative surveillance included routine clinical and laboratory examinations every third month, computed tomography scans of the abdomen, and radiographs of the chest every third month. After 5 years, the examination interval was extended to 12 months.

### Immunohistochemistry analysis

Immunohistochemical staining was carried out following the protocol of our previous study [16]. The primary antibody against L1CAM: mouse monoclonal antibody against the human ectodomain of L1 (UJ127, cat.^#^GTX72362; Gene Tex, Irvine, CA, dilution 1:500). The specificity of the primary antibody has been demonstrated by several previous studies [11,13,14]. Secondary antibody for the detection of primary antibody: anti-mouse IgG (Sigma, St. Louis, MO, USA). The negative controls were processed in a similar manner with PBS instead of primary antibody. Further, positive L1CAM expression confirmed by western blotting was used as positive controls for immunostaining.

Following a hematoxylin counterstaining, immunostaining was scored by two independent experienced pathologists, who were blinded to the clinicopathological parameters and clinical outcomes of the patients. The scores of the two pathologists were compared and any discrepant scores were trained through re-examining the stainings by both pathologists to achieve a consensus score. The number of positive-staining cells showing immunoreactivity on the membrane for L1CAM in ten representative microscopic fields was counted and the percentage of positive cells was calculated. The percentage scoring of immunoreactive tumor cells was as follows: 0 (0%), 1 (1-10%), 2 (11-50%) and 3 (>50%). The staining intensity was visually scored and stratified as follows: 0 (negative), 1 (weak), 2 (moderate) and 3 (strong). A final score was obtained for each case by multiplying the percentage and the intensity score. Therefore, tumors with a multiplied score exceeding 4 (median of total scores for L1CAM) were deemed to be low expressions of L1CAM; all other scores were considered to be high expressions of L1CAM.

### Western blot

The Western blot protocol and semiquantitative analysis were carried out following the protocol of Xu et al. [17]. L1CAM antibody (mouse monoclonal antibody, dilution 1:1000, Santa Cruz Biotechnology, Inc. USA) was used, and GAPDH antibody (CW0266, dilution 1:1,000, CoWin Biotech) was used as internal control.

### Quantitative RT-PCR

To measure the mRNA expression levels of L1CAM, total RNA was extracted from frozen liver tissues using TriZol reagent (Invitrogen) following the manufacturer’s instructions. Two micrograms of total RNA was subjected to reverse transcription to synthesize cDNA using the ProtoScript M-MuLV Taq RT-PCR Kit (New England Biolabs), according to the manufacture’s instruction, followed by real-time PCR using the TransStart Green qPCR SuperMix (TransGen Biotech). The primer sequences of L1CAM were forward primer, 5′- ACG AGG GAT GGT GTC CAC TTC AAA-3′, reverse primer, 5′- TTA TTG CTG GCA AAG CAG CGG TAG-3′. The transcription of GAPDH was used as an internal control for normalization. L1CAM expression levels were calculated relative to GAPDH using the delta-delta computed tomography method [18].

### Statistical analysis

The software of SPSS version13.0 for Windows (SPSS Inc, IL, USA) and SAS 9.1 (SAS Institute, Cary, NC) was used for statistical analysis. Fisher’s exact test and the X^2^ test were performed to assess associations between L1CAM expression and clinicopathological parameters. The Kaplan-Meier method was used for survival analysis, and differences in survival were estimated using the log-rank test. A multivariate survival analysis was performed for all parameters that were significant in the univariate analyses using the Cox regression model. Differences were considered statistically significant when *P* was less than 0.05.

## Results

### Expression of L1CAM protein and mRNA in HCC

To analyze the clinical value of L1CAM in HCC, we first evaluated its expression at protein and mRNA levels by immunohistochemical analysis, western blot analysis and quantitative RT-PCR.

**Figure 1:**
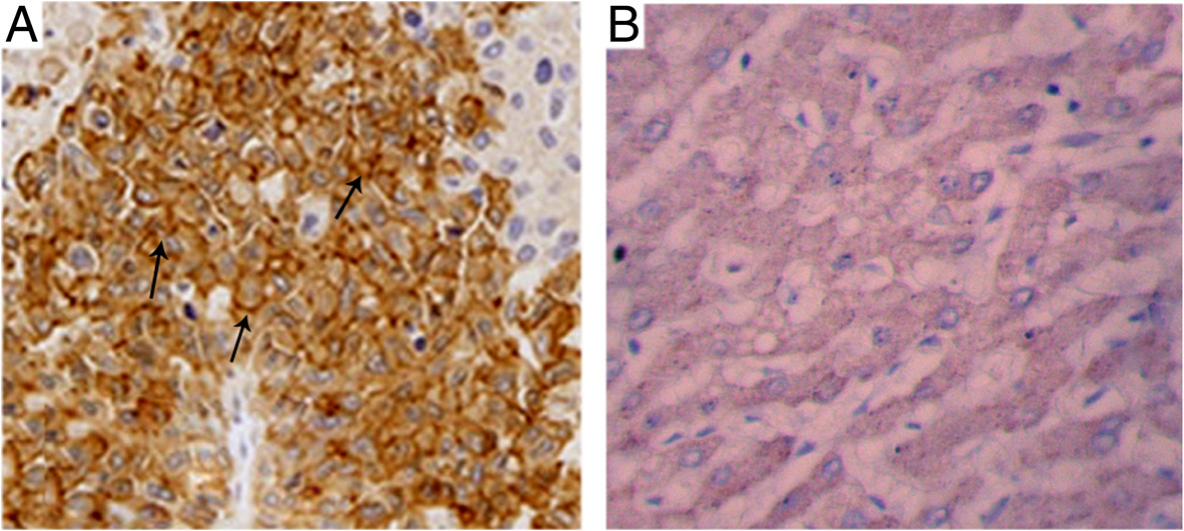
**Immunohistochemical staining of L1CAM expression in hepatocellular carcinoma (HCC) and adjacent nonneoplastic liver tissues (Original magnification × 400).A**, L1CAM positive staining was indicated by numerous yellowish granules in the membrane of HCC cells; **B**, L1CAM negative staining was seen in adjacent nonneoplastic liver tissues.

As the results, the immunostaining was homogeneous throughout the tumor. L1CAM immunostaining was mainly localized on the membrane of tumor cells of HCC tissues (Figure 1A). L1CAM expression was absent or sporadic in adjacent nonneoplastic liver tissues (Figure 1B). In addition, we found 82 (63.08%) of 130 HCC tissues with high L1CAM expression and 48 (36.92%) of 130 HCC tissues with low L1CAM expression, while all the adjacent nonneoplastic liver tissues with low L1CAM expression. Thus, the L1CAM immunostainings in HCC tissues were significantly higher than those in the adjacent nonneoplastic liver tissues (P <0.01).

**Figure 2:**
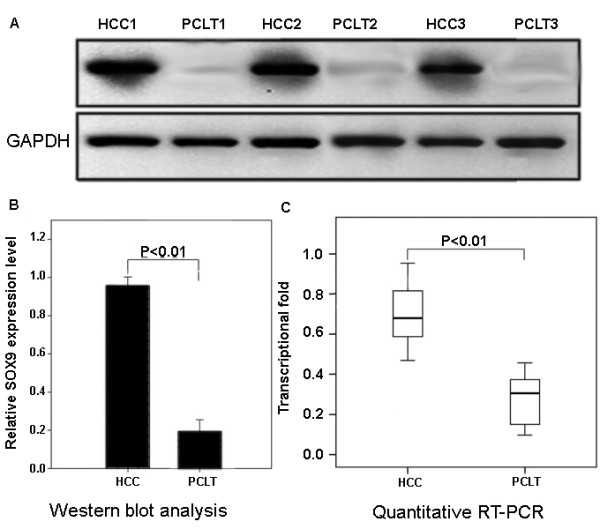
**Increased expression levels of L1CAM protein and mRNA in hepatocellular carcinoma (HCC) and adjacent nonneoplastic liver tissues.** (**A**) Representative Western blotting of L1CAM protein levels in HCC tissues and adjacent nonneoplastic liver tissues. (**B**) Semiquantitative Western blotting showed significantly increased L1CAM protein level in HCC tissues compared with adjacent nonneoplastic liver tissues. GAPDH was used as internal control. Means, standard deviation (SD), and P values were given (T test). (**C**) Significantly increased L1CAM mRNA level (P <0.01, Mann–Whitney test) in HCC tissues compared with adjacent nonneoplastic liver was detected by quantitative RT-PCR. GAPDH was used as internal control.

Additionally, Western blot analysis as an independent method was performed to confirm L1CAM protein expression. The distinct overexpression of L1CAM protein in HCC tissues compared with adjacent nonneoplastic liver tissues was also detected (P <0.01, Figure 2A and B), as well as significantly increased mRNA level by quantitative RT-PCR (P <0.01, Figure 2C).

### Association of L1CAM expression with the clinicopathological features of HCC

We next evaluated whether L1CAM protein expression was associated with clinicopathological features of patients with HCC by correlating immunohistochemical L1CAM staining results with T stage, tumor grade, presence of cirrhosis, underlying liver disease including alcohol abuse, viral hepatitis B and C, sex, and age (Table 1). As the results, we found that the high expression of L1CAM was significantly associated with advanced tumor stage (P = 0.02) and advanced tumor grade (P = 0.03), respectively.

### Prognostic values of L1CAM expression in HCC

**Figure 3:**
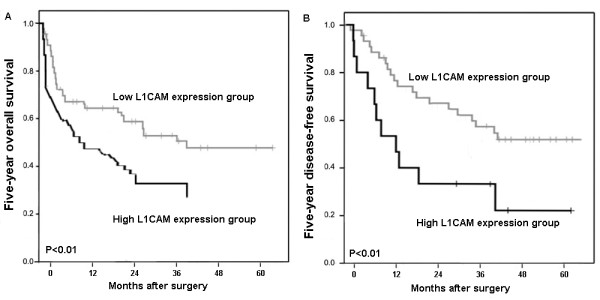
**Kaplan-Meier survival curves for L1CAM expression in the HCC patients.** The HCC patients with high L1CAM expression showed significantly shorter disease-free survival (P <0.01, **A**) and overall survival (P <0.01, **B**) rates than those with low L1CAM expression.

To further investigate the clinical usefulness of L1CAM expression in HCC, we compared five-year overall survival and five-year disease-free survival according to various clinicopathologic factors including the expression level of L1CAM. Five-year disease-free survival was observed in 30 (23.08%) patients, whereas in 100 (76.92%) patients, disease recurred, and 88 (67.69%) even died during a 5-year follow-up period. We observed a trend that 5-year disease-free survival in the group with high L1CAM expression was significantly poorer than that in the group with low L1CAM expression (P <0.01, log-rank test; Figure 3A). Additionally, the Kaplan-Meier plot of 5-year overall survival curves stratified by L1CAM expression was shown in Figure 3B. A significant relationship was found between L1CAM expression and 5-year overall survival (P <0.01, log-rank test, Figure 3B). Futhermore, in a multivariate Cox model, including tumor size, tumor stage, tumor grading, presence of cirrhosis, gender, age, and L1CAM staining, we found that L1CAM expression was an independent poor prognostic factor for both 5-year disease-free survival (hazards ratio [HR] = 2.279, 95% confidence interval[CI] = 1.185-5.697, P = 0.02, Table 2) and 5-year overall survival (HR = 3.269, CI = 1.136-7.328, P = 0.008, Table 2) in HCC.

**Table 2:** Multivariate survival analysis of five-year overall and disease-free survival in 130 patients with HCC

**Features**	**Five-year overall survival**			**Five-year disease-free survival**		
	**HR**	**95% CI**	**P**	**HR**	**95% CI**	**P**
**Age**	1.132	0.316-3.516	0.192	1.536	0.322-3.736	0.125
**Gender**	1.191	0.345-3.857	0.136	1.559	0.357-3.831	0.131
**Tumor size**	1.931	0.685-4.056	0.063	1.953	0.615-4.273	0.062
**Tumor stage**	2.879	1.366-5.196	0.009	2.686	1.386-6.009	0.01
**Tumor grade**	1.563	0.609-4.088	0.081	1.551	0.607-4.466	0.086
**Presence of cirrhosis**	1.919	0.738-4.102	0.063	1.921	0.793-4.219	0.062
**L1CAM expression**	3.269	1.136-7.328	0.008	2.279	1.185-5.697	0.02

## Discussion

In this study, we first dmonstrate that L1CAM protein and mRNA expression in human HCC tissue was significantly associated with tumor progression and clinicopathologic features. Immunohistochemical analysis of a large set of HCCs revealed that 63.08% of HCC were high expression for L1CAM. Notably, L1CAM immunoreactivity was distinctly increased in a substantial proportion of HCC cases compared with their adjacent nonneoplastic liver tissue, which was further confirmed by Western bloting analysis and Q-PCR analysis. Then, the expression of L1CAM in HCC tissues with advanced tumor stage and grade was significantly higher than that in early tumor stage and low tumor grade HCC, suggesting that L1CAM expression might be of clinical relevance in the aggressiveness of HCC. The impact of L1CAM expression on clinical outcome was assessed by Kaplan-Meier analyses. High L1CAM expression was associated with a significant trend toward both poorer disease-free and overall survival. Univariate and multivariate analyses clearly demonstrated that L1CAM expression was an independent risk factor predicting overall survival and disease-free survival of patients with HCC. The statistically significant impact of L1CAM expression for overall survival (P = 0.008) was more significant than the tumor stage (P = 0.009) that is widely used at present, suggesting that L1CAM expression could be a useful marker to predict patient survival.

It is necessary to identify biological markers associated with the advancement of tumor progression for early diagnosis of patients with aggressive tumors and poor prognosis, and for the development of new therapeutic strategies and the selection of the appropriate treatment. The cell dhesion molecule families, such as integrins, cadherins, immunoglobulin-like CAMs and selectins, are often aberrantly regulated in human malignancies, leading to the tumor progression [19]. L1CAM, a member of immunoglobulin-like CAMs, was first reported to be involved in human cancers by investigating its expression in B16 melanoma cells [20]. After that, L1CAM overexpression has been found in various other tumors. Especially in degestive system, the study of Issa et al. [21] observed that L1CAM expression was selectively enhanced on endothelium associated with pancreatic adenocarcinoma in situ and on cultured pancreatic tumor-derived endothelial cells *in vitro*; Kodera et al. [22] detected the expression of L1CAM in gastric cancer specimens, more often among the intestinal-type cancer, and further demonstrated the prognositc value of L1CAM expression in pT3-stage gastric cancer; Choi et al. [23] also indicated that L1CAM was not expressed in the normal epithelium of the gallbladder but in 63.8% of gallbladder carcinomas, remarkably at the invasive front of the tumors; regarding the clinical significance, they demonstrated that L1CAM expression was significantly associated with the aggressiveness and poor prognosis of gallbladder carcinomas. With the similar results of these previous studies, our data also shown the association of L1CAM overexpression with the advancement and short survival of HCC patients.

The function of L1CAM determines its contribution to the tumorigensis. At first, L1CAM is a target gene of β-catenin-TCF signaling, which is an important cancer-related pathway. Many β-catenin target genes including metalloproteases, cell-extracellular matrix components, transcription factors, and cell adhesion molecules have been demonstrated to be involved in later stages of tumorigenesis that can confer invasive and metastatic capacities [24]. In 2006, Huszar et al. [25] identified L1CAM as a novel target gene of β-catenin-TCF signaling which is implicated in human colon cancer development. LEF/TCF binding sites were detected in the L1CAM promoter and an inducible dominant negative TCF, or an siRNA to β-catenin, suppressed the expression of L1CAM in colon cancer cells [26]. In addition, L1CAM induces ERK activation and ERK-regulated genes, including various integrin genes associated with cell motility and invasion. It was linked to activation of ERK and focal adhesion kinase to apoptosis protection in ovarian carcinoma [27]. Thirdly, L1CAM does not only mediate homophilic binding between cells, but also forms heterophilic interactions with various ECM proteins and their receptors. The L1CAM-induced cell motility was shown to involve a direct interaction of the shed L1CAM ectodomain, or the full-length L1CAM, with integrins, implying that L1CAM may play a role in cancer promotion and metastasis by also mediating cell-ECM interactions [28]. Because of its involvement in a wide variety of human cancers, L1CAM has been considered as a target molecule for cancer therapeutics. For example, the study of Bao et al. [29] found that L1CAM is required for maintaining the growth and survival of CD133+ glioma cells both *in vitro* and *in vivo*, and L1CAM may represent a cancer stem cell specific therapeutic target for improving the treatment of malignant gliomas and other brain tumors; Hung et al. [30] reported that targeting L1CAM using lentivirus-mediated shRNA may be a useful molecular pharmaceutical approach for the treatment of advanced oral squamous cell carcinoma; These previous studies suggested that using L1CAM as a drug target might improve the cancer patients’ outcome. The value of L1CAM in HCC therapy also needs further evaluation.

In conclusion, our data suggest that L1CAM is overexpressed in HCC tissues compared with their benign counterparts. To the best our knowledge, this is the first study evaluating the expression levels of L1CAM mRNA and protein in HCC tissues and its association with clinicopathologic parameters. Especially, the most important finding of this study is that L1CAM also is a novel and potential factor for predicting the poorer prognosis of HCC patients after surgery.

## Competing interests

The authors declare that they have no competing interests.

## Authors’ contributions

XDG and LX carried out the experimental studies and drafted the manuscript. LZ, TS, JZ, and HWL carried out part of the experimental studies. RYP and JMZ designed the experiments and modified the manuscript. All authors read and approved the final manuscript.
